# The Continuing Impact of Oncology Drug Shortages

**Published:** 2013-03-01

**Authors:** Pamela Hallquist Viale

Oncology drug shortages remain prevalent, and as advanced practitioners, we are continuing to experience this troubling phenomenon. We introduced this important issue previously in the July/August 2011 issue of the *Journal of the Advanced Practitioner in Oncology*. We noted that the number of shortages has increased dramatically in the past decade and that this situation is an ongoing challenge for advanced practitioners (Vogel & Ervin, 2012). There are a myriad of causes for the shortages; the leading reasons cited are problems with manufacturing facilities (43%), interruptions or delays in the manufacturing or shipping of drugs (15%), and the lack of availability of key active pharmaceutical ingredients (10%; Printz, 2012). There is no doubt that because of drug shortages, the potential exists for negative effects in the care of oncology patients.

Many of these agents are generic drugs (often injectables) that have been used for decades in the treatment of childhood leukemia and curable cancers (Rochon & Gurwitz, 2012; Gatesman & Smith, 2011). Unfortunately, without adequate financial compensation, some manufacturers stop making generic drugs (Gatesman & Smith, 2011). What’s more, if more expensive brand name drugs that provide a higher profit margin for the manufacturer are available, production of the generic drug may cease permanently (Gatesman & Smith, 2011).

## New Evidence Linking Drug Shortages to Poor Outcomes

*The New England Journal of Medicine* recently published a report on the impact of drug shortages on children with cancer (Metzger, Billet, & Link, 2012). Mechlorethamine (nitrogen mustard) is an essential agent used in the treatment of Hodgkin lymphoma; this agent, one of our oldest therapeutic drugs, has been in use for over 5 decades (Metzger, Billet, & Link, 2012). In 2002, the Pediatric Hodgkin Lymphoma Consortium switched to the use of the Stanford V regimen from the previous standard of mechlorethamine, vincristine, procarbazine, and prednisone (MOPP). Stanford V includes mechlorethamine, vinblastine, doxorubicin, vincristine, bleomycin, etoposide, and prednisone given in a shorter course of therapy without procarbazine, helping to preserve fertility and reduce the risk of secondary leukemia and other side effects as seen with the use of MOPP (Metzger, Billet, & Link, 2012).

When mechlorethamine was in short supply in 2009, the Consortium decided to substitute cyclophosphamide based on a review of the literature suggesting the substitution was safe, although no randomized study had demonstrated equivalence in efficacy (Metzger, Billet, & Link, 2012).

The authors of the study compared the probability of event-free survival among 181 patients treated with the original Stanford V regimen vs. the modified regimen using cyclophosphamide. The retrospective comparison demonstrated that substituting cyclophosphamide for mechlorethamine was significantly less effective, with a 2-year event-free survival of 75% for the cyclophosphamide patients vs. 88% for the mechlorethamine patients. As none of the patients in the study has died, a survival difference cannot yet be ascertained. But the treatment for patients who relapsed included more toxic salvage therapies with intensive cytoreduction followed by autologous stem-cell transplantation, therapies that carry significantly more side effects (Metzger, Billet, & Link, 2012).

Since approximately 80% of children with cancer can potentially be cured of their disease, shortages of vital therapies can have significant and potentially devastating effects on our patients (Metzger, Billet, & Link, 2012). Substitutions of different therapies for those treatments unavailable due to manufacturing or other problems may produce negative effects on outcomes for patients, as evidenced by the study discussed here.

## FDA Approval of Generic Doxil

In a move designed to address the oncology drug shortages and help patients get needed therapies as soon as possible, the US Food and Drug Administration (FDA) is using a system to make the review process of generic medications move more quickly. A generic version of Doxil (liposomal doxorubicin), a drug currently on the shortage list, has just been approved (Clarke, 2013). This development will help meet the demand for Doxil. (The generic form of Doxil, which contains the same active ingredient as the branded version, had not previously been approved in the United States.) Approval of the new generic form of Doxil represents enforcement discretion by the FDA for the present time, although approval could change in the future (FDA, 2013).

## Taking Action

The Metzger et al. study previously discussed represents the first objective evidence that substitution based on drug shortages can lead to a significant change in event-free survival. When oncology advanced practitioners sit on hospital committees struggling with action plans to combat drug shortages, the negative outcomes seen in the Metzger et al. study should be referenced. The authors of a recently published paper on the ethical aspects of managing drug shortages have recommended that management of shortages include standard of care guidelines to ensure that all patients receive the appropriate therapy for their disease (Valgus, Singer, Berry, & Rathmell, 2013).

Drug substitution may have a more significant effect on patient outcomes than previously thought and should be considered when developing guidelines. Unfortunately, oncology drug shortages have become routine. Standard of care guidelines may provide a framework to help manage this critical problem in oncology care.
